# Consumption of an Anthocyanin-Rich Extract Made From New Zealand Blackcurrants Prior to Exercise May Assist Recovery From Oxidative Stress and Maintains Circulating Neutrophil Function: A Pilot Study

**DOI:** 10.3389/fnut.2019.00073

**Published:** 2019-05-29

**Authors:** Roger D. Hurst, Kirsty A. Lyall, Joanna M. Roberts, Anton Perthaner, Robyn W. Wells, Janine M. Cooney, Dwayne J. Jensen, Natalie S. Burr, Suzanne M. Hurst

**Affiliations:** ^1^The New Zealand Institute for Plant and Food Research Ltd., Palmerston North, New Zealand; ^2^The New Zealand Institute for Plant and Food Research Ltd., Hamilton, New Zealand; ^3^AgResearch Ltd., The Hopkirk Research Institute, Palmerston North, New Zealand

**Keywords:** anthocyanin bioavailability, pilot exercise intervention study, oxidative stress, neutrophil phagocytosis, exercise recovery

## Abstract

**Aim:** To evaluate blackcurrant anthocyanin-rich extract (BAE) consumption on time- and dose-dependent plasma anthocyanin bioavailability and conduct a pilot study to explore the potential effect of BAE in promoting recovery from exercise-induced oxidative stress, and maintenance of circulating neutrophil function.

**Methods:** Time- and dose-dependent blackcurrant anthocyanin bioavailability was assessed using LC-MS in 12 participants over 6 h after the ingestion of a placebo or BAE containing 0.8, 1.6, or 3.2 mg/kg total anthocyanins. In a separate pilot intervention exercise trial, 32 participants consumed either a placebo or 0.8, 1.6, or 3.2 mg/kg BAE (8 individuals per group), and then 1 h later performed a 30 min row at 70% VO_2_max. Blood was collected during the trial for oxidative, antioxidant, inflammatory, and circulating neutrophil status.

**Results:** Consumption of BAE caused a time- and dose-dependent increase in plasma anthocyanins, peaking at 2 h after ingestion of 3.2 mg/kg BAE (217 ± 69 nM). BAE consumed 1 h prior to a 30 min row had no effect on plasma antioxidant status but hastened the recovery from exercise-induced oxidative stress: By 2 h recovery, consumption of 1.6 mg/kg BAE prior to exercise caused a significant (*P* < 0.05) 34 and 32% decrease in post-exercise plasma oxidative capacity and protein carbonyl levels, respectively, compared to placebo. BAE consumption prior to exercise dose-dependently attenuated a small, yet significant (*P* < 0.01) transient 13 ± 2% decline in circulating neutrophils observed in the placebo group immediately post-exercise. Furthermore, the timed consumption of either 1.6 or 3.2 mg/kg BAE attenuated a 17 ± 2.4% (*P* < 0.05) decline in neutrophil phagocytic capability of opsonised FITC-*Escherichia coli* observed 6 h post-exercise in the placebo group. Similarly, a dose-dependent increase in neutrophil surface expression of complement receptor-3 complex (CR3, critical for effective phagocytosis of opsonised microbes), was observed 6 h post-exercise in both 1.6 and 3.2 mg/kg BAE intervention groups.

**Conclusions:** Consumption of BAE (>1.6 mg/kg) 1 h prior to exercise facilitated recovery from exercise-induced oxidative stress and preserved circulating neutrophil function. This study provides data to underpin a larger study designed to evaluate the efficacy of timed BAE consumption on post-exercise recovery and innate immunity.

## Introduction

Whether an athlete is undertaking an intense training program for a sporting event, or is a recreational exerciser just trying to keep fit and healthy, there is a fine balance between achieving the maximum health benefits of exercise and the accumulation of tissue damaging oxidative stress. Exercise-induced reactive oxygen (ROS) and nitrogen species (RNS) are unavoidable by-products of increased oxidative metabolism. Although initially thought to be detrimental to health (1), ROS/RNS have recently been shown to play a pivotal role in the body's adaptive response to exercise (2, 3) as blocking ROS/RNS by high doses of dietary antioxidants eliminates the health promoting effects of exercise (4, 5). ROS/RNS released during exercise serve as signaling molecules and act at various cellular transcription factors (by direct oxidation or through reversible interactions with upstream redox-sensitive proteins) to regulate numerous cellular innate defense systems, including mitochondrial adaptation to enhance antioxidant capacity (6), and modulation of immune status (7). Moreover, whilst regular exercise boosts the body's natural defenses overall (8–10), studies have shown a transient drop in circulating leukocyte number and/or function following exercise. This is driven by an oxidative imbalance that is dependent upon the exercise intensity and duration as well as the fitness of the individuals, which can create an “open window” for opportunistic infections (10–12). Circulating neutrophils are the first line of defense against microbial infection, and are instrumental in coordinating acute inflammatory, tissue repair and appropriate adaptive immune responses (13). Therefore, it is important that the number and functionality of circulating neutrophils are maintained during exercise and recovery to preserve immune defenses. Under most exercise conditions, oxidative balance is maintained within physiological limits minimizing the potential for oxidative damage and increased susceptibility to infection (2, 10). However, prolonged intense exercise in the absence of sufficient recovery time can result in oxidative stress leading to immune fatigue, delayed tissue repair/adaptation, enhanced susceptibility to infection, and ultimately immune suppression and loss of performance (11). Foods and/or dietary supplements that support innate immunity (including maximizing cellular antioxidant capacity) may serve to reduce the possible detrimental effects of exercise training and promote exercise-induced physiological adaptation.

Implementation of an appropriate nutritional strategy is integral to boosting the adaptive effects of regular exercise (12, 14). Functional nutritional strategies show that consumption of carbohydrate and/or protein post-exercise not only replenishes glycogen stores, but also improves skeletal muscle strength and ergonomics (15, 16). Recently, the potential bioactive efficacy of dietary micronutrients, vitamins, minerals, and plant extracts within an exercise regime has been explored (12, 17, 18). The primary focus has been on tailoring micronutrient bioactivity to the needs of the athlete or recreational exerciser, such as speeding up recovery between exercise bouts or maintaining immune defenses to avoid illness. Dietary antioxidants, such as vitamin C and E, are often taken during exercise training to reduce exercise-related oxidative stress damage (19, 20). However, while these are an essential component of daily dietary intake, and have been shown to prevent protein oxidation during exercise, their suitability for the prevention of exercise-induced oxidative stress is questionable. Nutritional intervention with high strength antioxidants have been shown to prevent the adaptive action of ROS/RNS and should be avoided as a post-exercise recovery strategy (2). Select plant-derived polyphenolic compounds show a high endogenous antioxidant capacity (21), and numerous *in vitro* cell studies have demonstrated the inherent antioxidant properties of polyphenolic compounds, including anthocyanins (22, 23). However, nutritional intervention studies with dietary polyphenolic-rich foods/supplements have found that the attenuation of exercise-induced oxidative stress, exercise recovery and adaptation events was independent of their direct antioxidant properties (17, 24, 25). Furthermore, daily consumption of foods, drinks and/or supplements rich in polyphenolic compounds have been shown to support innate immune defenses and ameliorate metabolic-related (as well as delay the onset of age-related) chronic inflammatory illnesses (26, 27). Nutritional intervention has also been successfully employed to reduce post-exercise susceptibility to infection in athletes undertaking demanding training schedules (2). However, the efficacy of timed polyphenolic rich supplementation to support sports training adaptation and performance benefits is unclear.

Blackcurrants (*Ribes nigrum*) possess a high inherent antioxidant capacity compared to most other fruits, primarily due to their high anthocyanin and vitamin C content (28, 29). Recent studies, however, show that the inherent chemical antioxidant properties of fruits is not necessarily a good indicator of their actual health-benefiting properties. Blackcurrant anthocyanins, for example, have also been shown to exhibit health properties independent of their antioxidant capability using both *in vitro* cell studies (30–32) and human nutrition studies, whereby consumption of blackcurrant anthocyanin compounds have been reported to support innate immunity (33–35), improve eye blood flow (36) and augment the adaptive events of exercise (37, 38). In this study, we assessed the dose and temporal bioavailability of anthocyanins after consumption of a New Zealand blackcurrant anthocyanin-rich extract (BAE) and apply this knowledge in a pilot trial designed to examine the dose-dependent effect of pre-exercise BAE consumption on recovery from exercise-induced oxidative stress and the maintenance of circulating neutrophil function.

## Materials and Methods

### Human Trial Design

#### Subject Selection

The human trials conducted in this study were approved by either the Northern (NTY/09/10/105, Hamilton) or Central (CEN/09/11/088, Wellington) Ethical Regional Committees of New Zealand. Healthy individuals between 20 and 60 years old were recruited from Hamilton and Palmerston North communities and within The New Zealand Institute for Plant and Food Research Ltd. All subjects provided written informed consent and refrained from (i) eating foods (including drinks) containing anthocyanins and berryfruits and (ii) taking antioxidant dietary supplements 24 h prior to the start of the trials. Participants recruited for the pilot intervention exercise trial, exercised daily, displayed similar physical characteristics and habitual activity scores (Table 1); assessed by Baêcke questionnaire (39). In addition, the 70% VO_2_max exercise intensity used in this pilot trial has successfully been shown by us to evoke a measureable increase in oxidative stress (33, 40): The 70% VO_2_max exercise intensity was achieved by participants rowing at a pace that raised their heart rate to the equivalent of their predicted 70% VO_2_max (41, 42) through the monitoring of their heart rate using a heart monitor (model AXN700 Polar Electro, Auckland, New Zealand) for the duration of the 30 min row. Subjects were excluded from the study if they had known fruit allergies, blood borne diseases (e.g., hepatitis), viral or bacterial illness, were under-going an immunization programme, taking mediation that affected blood properties (e.g., clotting), pregnant, or planning to get pregnant. Individuals participating in the exercise trial were also excluded if they were unable to perform the 30 min rowing exercise (e.g., current injury or recovering from injury received within the last 3 months) or did not meet the required fitness criteria.

**Table 1:** Subject characteristics and habitual activity.

**Nutritional intervention**	**Placebo**	**0.8 mg/kg**	**1.6 mg/kg**	**3.2 mg/kg**	
**Variable**					
Age (yr)	42 ± 9	44 ± 11	44 ± 11	44 ± 11	(*P* = 0.97)
Weight (kg)	84 ± 19	73 ± 12	82 ± 19	75 ± 12	(*P* = 0.42)
Height (cm)	178 ± 6	170 ± 2	174 ± 7	174 ± 8	(*P* = 0.18)
**Bioimpedance Measures**					
Soft lean mass (kg)	64.3 ± 19.0	53.8 ± 8.5	60.9 ± 11.4	58.4 ± 9.8	(*P* = 0.17)
Body fat mass (kg)	15.7 ± 9.3	21.6 ± 18.6	17.6 ± 10.0	19.5 ± 18.7	(*P* = 0.85)
Percent body fat (%)	17.7 ± 5.6	20.8 ± 7.6	20.4 ± 7.4	17.5 ± 5.8	(*P* = 0.61)
Body mass index (kg/m2)	26.5 ± 4.8	25.3 ± 2.7	27.0 ± 4.9	24.8 ± 2.0	(*P* = 0.59)
**Habitual Activity Scores**					
Work *(max. 10)*	4.4 ± 0.7	4.4 ± 0.7	4.3 ± 0.7	4.5 ± 0.6	(*P* = 0.94)
Sports *(max. 8)*	5.0 ± 1.2	4.6 ± 1.6	5.5 ± 0.8	4.2 ± 1.6	(*P* = 0.25)
Leisure *(max. 15)*	6.9 ± 2.6	5.2 ± 2.3	6.1 ± 2.3	5.7 ± 3.0	*(P* = 0.55)

*Results are expressed as mean ± SD of n = 8 subjects per nutritional intervention group*.

#### Nutritional Intervention

The blackcurrant anthocyanin-rich extract (BAE) consisted of 34% anthocyanins and can be sourced from the New Zealand Blackcurrant Co-operative (www.nzblackcurrants.com/, New Zealand). The dietary extract was made from New Zealand blackcurrants and displayed a high inherent antioxidant capacity (6424; ORAC and 3810; FRAP μmol Trolox equivalents) and contained < 1 mg/100 g of vitamin C. Administered BAE (0.8, 1.6, or 3.2 mg/kg) amounts were calculated using participant's weight (kg) and total anthocyanins (mg) measured by LC-MS. A fruit sugar placebo (PLA) similar to that used in previous nutrition exercise intervention trials was selected.

#### Study Format (Figure 1)

- *Plasma anthocyanin bioavailability trials*. All trials were performed at the same time of day (morning ~8 am) and 2 h after eating a set breakfast consisting of One-Square Meal^TM^ (Queenstown, New Zealand) cereal bar and drink (water). The trial design for time-dependent plasma anthocyanin profiles was a non-blinded, single arm format recruiting 12 individuals (6 female and 6 males). Trial participants donated blood samples before consuming two opaque gelatine capsules containing BAE (3.2 mg/kg total anthocyanins). Further blood samples were taken at 0.5, 1, 2, and 6 h post ingestion. To examine the dose-dependent plasma bioavailability of blackcurrant anthocyanins, a non-blinded parallel study design was employed where 24 participants were randomly assigned into one of four arms (6 individuals per arm): placebo (PLA) or BAE (0.8, 1.6, or 3.2 mg/kg total anthocyanins). A blood sample was taken prior to participants consuming 1–2 opaque gelatine capsules and then 1 h later.
- *Pilot study. BAE intervention exercise recovery*. A double-blind placebo-controlled parallel trial design consisted of four arms: PLA or BAE at 0.8, 1.6, or 3.2 mg/kg total anthocyanins. Thirty-two trial participants were randomly assigned to one of the four arms (8 individuals per arm) by an independent researcher and blinded from both trial participants and the trial coordinator. This trial was performed in the morning and all participants prepared for the trial as described above. At the start of the trial, a blood sample was taken and participants were fitted with a heart monitor and asked to consume 1-2 gelatin capsules with water. After 1 h, participant's heart rate was recorded and another blood sample collected, participants then performed a 30 min row (using a Concept 2 rower) at their predicted 70% VO_2_max (see methods). Upon completion, a blood sample was taken and the distance (meters) rowed and participants heart rates were recorded. Further blood samples were collected during post-exercise recovery at 2 and 6 h.

**Figure 1:**
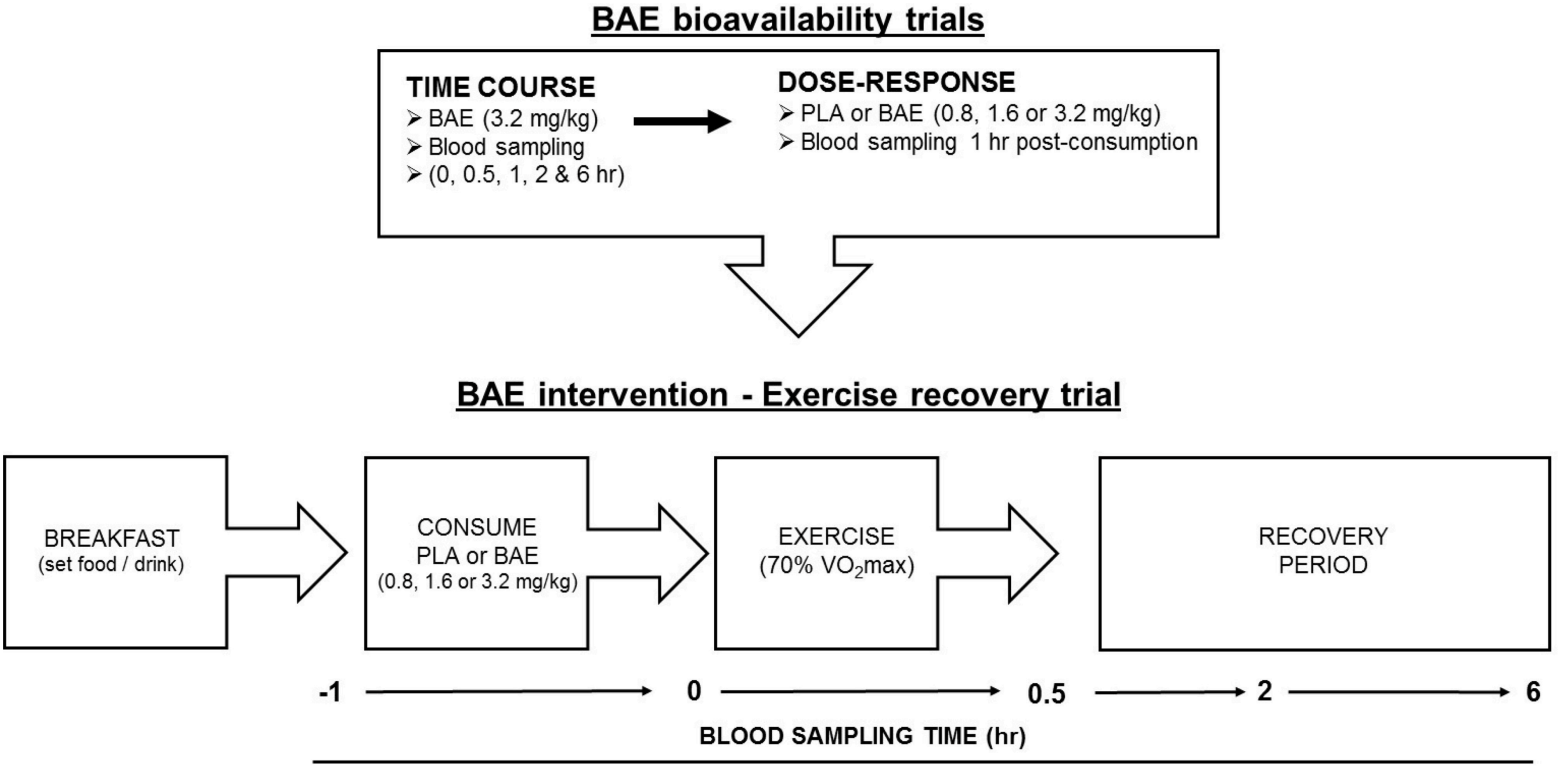
Flow chart of study design involving blackcurrant anthocyanin-rich extract (BAE) bioavailability (time course and dose-response) trials and BAE intervention - exercise recovery trial. PLA = placebo.

#### Blood Processing

- *Bioavailability trials*. Collected blood samples were immediately centrifuged (300 g, room temperature [RT], 10 min) and the plasma acidified with 5% trifluoroacetic acid and stored at −80°C until liquid chromatography-mass spectrometry (LC-MS) analysis.
- *Intervention trial*. Blood collected was used to assess blood lactate (Arkray Lactate biosensor Pro™ 2, Baden, Swizerland), circulating neutrophil status, or centrifuged (300 *g*, RT, 10 min) and the plasma frozen at −80°C for future measurement of oxidative, antioxidant, and inflammatory indices.

### Plasma Biochemical Measures

#### Anthocyanin Bioavailability

Plasma anthocyanins were measured by LC-MS analysis as previously described by Watson et al. (43). Malvidin-3-galactoside (not present in blackcurrants) was used as an internal standard (IS) and data was calculated as total anthocyanins nM_equ_.

#### Oxidative Stress Parameters

Plasma oxidative capacity and protein carbonyl levels were used to evaluate exercise-induced oxidative stress.

- *Oxidative generating capability* was assessed using a fluorescence kinetic assay previously described (40). Data were presented as a change in fluorescence intensity (FI) after 5 min (ΔFI_5min_).
- *Protein carbonyls* were measured using the modified version of a colorimetric end-point assay previously described (44). Carbonyl levels were calculated as nmol/mg protein.

#### Antioxidant Measures

Antioxidant capacity was assessed using ferric reducing ability of plasma (FRAP) (45). FRAP was measured against a standard curve of Trolox (Merck NZ), calculated as μM Trolox equivalents and data presented as % pre-exercise values.

#### Systemic Inflammatory Indices

Plasma C-reactive protein (CRP) was assessed using a specific CRP ELISA kit (R&D systems, Pharmaco, Auckland, New Zealand), calculated as mg/L and presented as % pre-exercise values.

### Neutrophil Characteristics and Function

#### Preparation of Blood Samples

- *Circulating blood neutrophil levels*. Collected blood was lysed (BD-FACS erythrocyte lysis buffer, BD, BioScience, Auckland, New Zealand) and incubated with FITC-CD18 antibodies (BioLegend, San Diego, USA) at RT (in the dark) for 15 min, washed twice and re-suspended in PBS.
- *CR3 surface receptor expression*. Erythrocyte-lysed blood samples were incubated with FITC-CD18 or PE/Cy7-CD11b antibodies (BioLegend) at RT (in the dark) for 15 min, washed twice and re-suspended in PBS.
- *Phagocytic capability*. Neutrophil phagocytosis activity was assessed using a modified method described by White-Owen et al (46). Blood samples were incubated with opsonised FITC-conjugated *E. coli* (Fluorescent BioParticles®, Molecular Probes, Life Technologies) at 37°C for 5 min, erythrocytes lysed and leukocytes incubated with propidium iodide for 10 min. Non-ingested FITC-*E. coli* were quenched with 10% trypan blue solution immediately prior to flow cytometry analysis.

#### Flow Cytometry Analysis

All blood samples were prepared for flow cytometry analysis within 2 h of collection. Flow cytometry was performed using a BD FACSCalibur running CellQuest PRO equipped with 488 and 633 nm lasers and four fluorescence detectors. Data files were analyzed following acquisition using FlowJo analysis software as described below

- *Circulating blood neutrophil levels*. Using an initial leukocyte forward (FSC) vs. side (SSC) scatter plot, FITC-CD18 labeled granulocytes were identified (~ 70% of total leukocyte population). Circulating neutrophil levels were then calculated as a % of CD18 positive neutrophils (CD18^+^) and presented as (a) a single cell scatter plot or (b) expressed as % pre-exercise values in response to exercise and recovery.
- *CR3 surface receptor expression*. Scatter plots were created to capture CD11b and CD18 expression and used to evaluate changes in combined CD11b/CD18 (CR3^+^) surface co-expression. CR3^+^ surface expression on circulating neutrophils were then calculated as % CR3^+^within a low-moderate and tight high gate (labeled as CR3^+high^) neutrophil CR3^+^ population. Results are presented as (a) scatter plots highlighting the two distinct CR3^+^ neutrophil populations or (b) expressed as % CR3^+high^ neutrophils.
- *Phagocytic capability*. Application of a scatter plot of blood granulocyte population enabled the selection of FITC positive and negative gates in each subject to determine the shift in FITC-*E. coli* positive neutrophil population and hence neutrophil phagocytic activity. Results are calculated as (a) scatter plot showing % FITC-*E. coli* positive and negative neutrophil populations or (b) as % FITC-*E. coli* positive neutrophils presented as % pre-exercise values.

### Statistical Analysis

A one-way ANOVA was applied to analyse the time-dependent plasma anthocyanin bioavailability data sets to examine statistical significance of time within specific individuals. This type of analysis was also applied when analyzing differences in subject physical characteristics and Baêcke questionnaire typical activity scores. Data sets examining multiple comparisons (i.e., dose-dependent plasma anthocyanin bioavailability and nutritional intervention exercise) were assessed by a two-way ANOVA, followed by *post hoc* paired Student's *t*-test analysis where appropriate. Comparison between two groups was assessed using a paired Student's *t*-test. In addition, the original data were transformed (where appropriate) to achieve normality and constant variance in the residuals prior to statistical analysis. Statistical significance for all indices was set at *P* < 0.05 with a confidence level of 95%.

## Results

### New Zealand Blackcurrant Anthocyanin Bioavailability Profiles

#### Time-Dependent Plasma Anthocyanin Bioavailability

**Figure 2:**
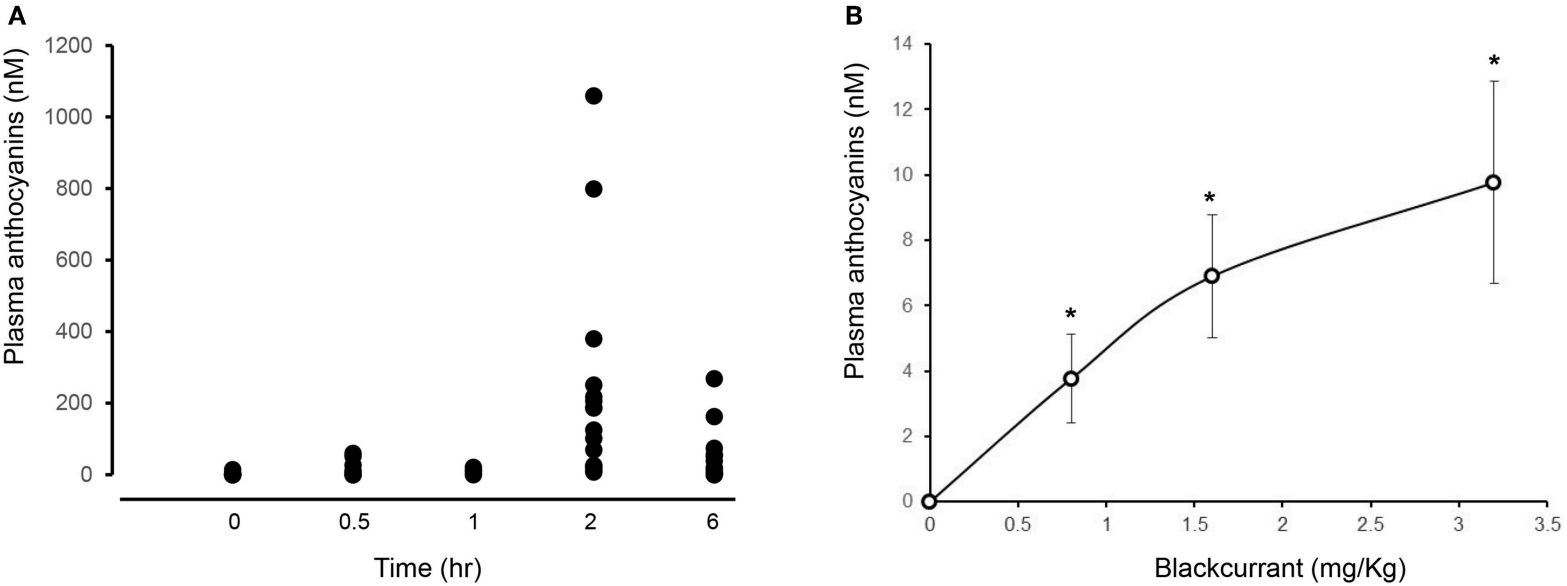
Impact of time **(A)** and dose **(B)** of blackcurrant anthocyanin-rich extract (BAE) consumption on plasma anthocyanins. Results are expressed as mean ± SEM of plasma total anthocyanins (nM). ^*^P < 0.05 represents a statistical difference from plasma anthocyanin levels at either **(A)** time zero or **(B)** the placebo group.

All participants showed an increase in plasma anthocyanins as early as 30 min after consuming 3.2 mg/kg BAE (14.6 ± 6.6 nM). This peaked after 2 h, displaying a plasma anthocyanin concentration ranging from 11 to 1,059 nM (Figure 2A).

#### Dose-Dependent Plasma Anthocyanin Bioavailability

A dose-dependent increase in plasma anthocyanins was observed 1 h after the consumption of BAE (Figure 2B): Participants who had consumed BAE containing 0.8 (3.7 ± 1.3 nM), 1.6 (6.9 ± 1.8 nM), or 3.2 (9.8 ± 3.1 nM) mg/kg anthocyanins showed a significant (*P* < 0.05) increase in plasma anthocyanins compared to the PLA (0.1 ± 0.3 nM).

Based upon these plasma blackcurrant anthocyanin bioavailability trials, we selected a 1 h pre-feeding intervention strategy, in a pilot trial, to explore the influence of pre-exercise BAE consumption on exercise-induced changes in oxidative stress and innate immunity during recovery.

### Exercise Physical Indices, Systemic Antioxidant, and Inflammation Status

#### Exercise Physical Outcomes

**Figure 3:**
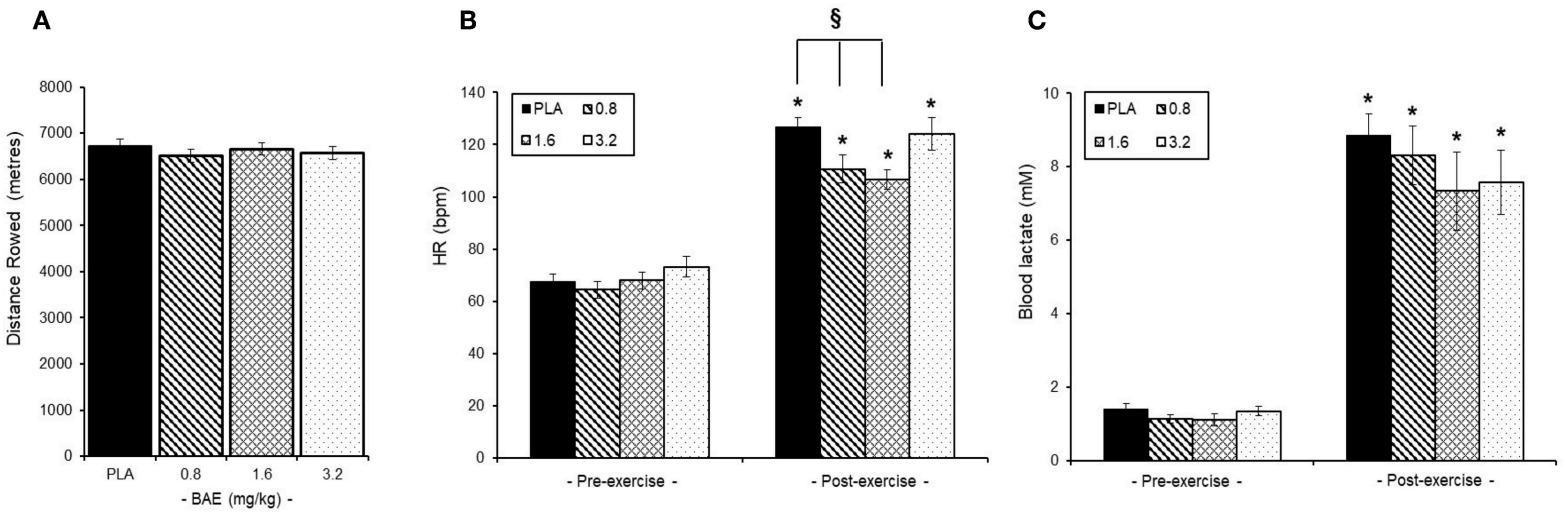
Influence of blackcurrant anthocyanin-rich extract (BAE, mg/kg) consumption 1 h prior to exercise on performance **(A)**, heart rate **(B)**, and blood lactate levels **(C)**. Results are mean ± SEM, *n* = 8 individuals per group. ^*^P < 0.05 represents a statistical difference from pre-exercise values. ^§^*P* < 0.05 represents a statistical difference from placebo for the groups highlighted.

No overall difference was observed in the distance rowed by the trial participants irrespective of intervention group (Figure 3A). Similarly, pre-exercise consumption of BAE had no effect on either baseline heart rates (HR, Figure 3B) or blood lactate (Figure 3C) levels. However, post-exercise HR were significantly (*P* < 0.05) lower in participants who had consumed 0.8 (126.9 ± 3.5 bpm) or 1.6 (106.6 ± 3.6 bpm) mg/kg BAE compared to the PLA (126.9 ± 3.5 bpm). Furthermore, pre-exercise consumption of 1.6 or 3.2 mg/kg BAE caused a marginal, yet non-significant, 9 and 7% decrease in post-exercise blood lactate levels (Figure 3C); 7.3 ± 1.1 mM (*P* = 0.16) or 7.5 ± 0.8 mM (*P* = 0.12), respectively.

#### Systemic Antioxidant and Inflammation Status

**Figure 4:**
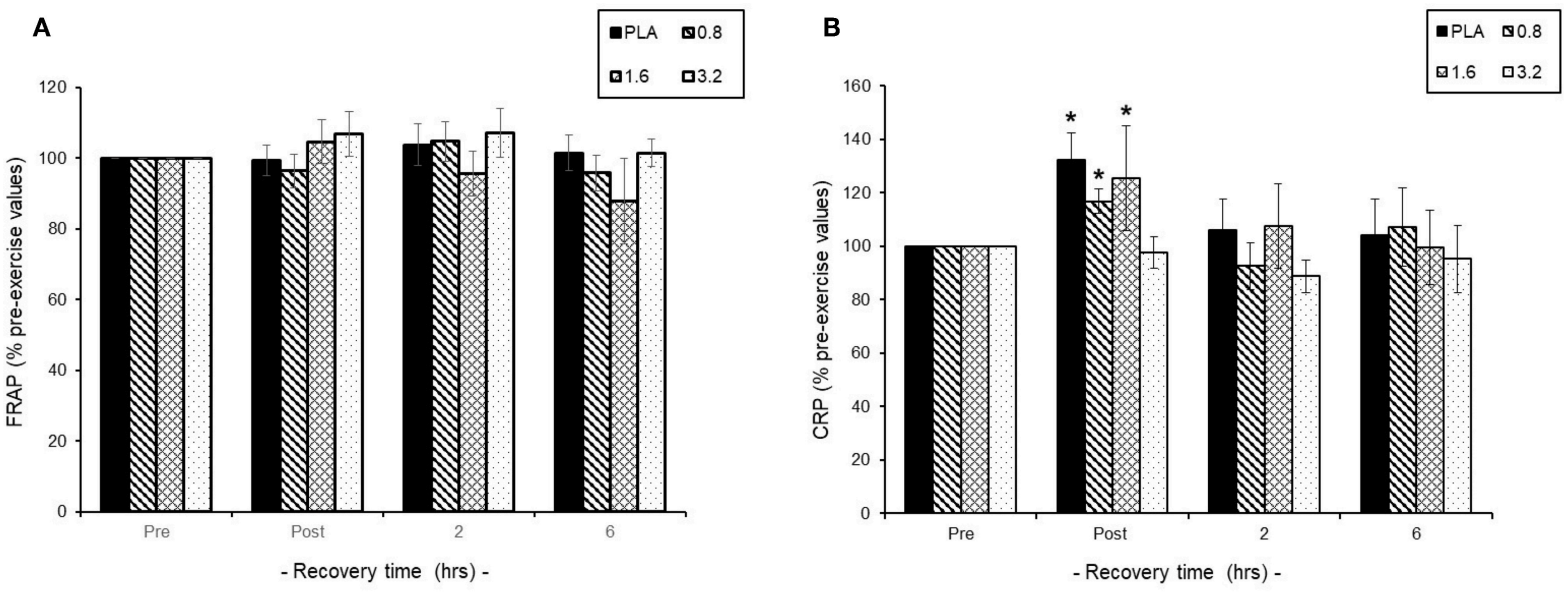
Effect of blackcurrant anthocyanin-rich extract (BAE, mg/kg) consumption 1 h prior to exercise on **(A)** plasma ferric reducing ability of plasma (FRAP) and **(B)** C-reactive protein (CRP). Results are expressed as mean ± SEM % pre-exercise values, *n* = 8 individuals per group. ^*^P < 0.05 represents a statistical difference from pre-exercise CRP levels, respectively, within each treatment group.

Pre-exercise consumption of BAE had no effect on baseline plasma FRAP or CRP levels and so exercise-induced changes were expressed as % pre-exercise (Figures 4A,B). We found that 30 min exercise in the presence or absence of BAE intervention had no impact upon plasma antioxidant (FRAP) status during exercise recovery (Figure 4A). The 30 min row evoked a transient plasma CRP increase (*P* < 0.05) in participants who had consumed PLA (1.3 ± 0.7 mg/L), 0.8 (1.4 ± 0.8 mg/L), or 1.6 (1.1 ± 0.4 mg/L) mg/kg BAE, which returned to pre-exercise levels after 2 h post-exercise recovery. No change in plasma CRP levels were observed in participants who had consumed 3.2 mg/kg BAE 1 h prior to the exercise.

### Recovery From Exercise-Induced Oxidative Stress

#### Plasma Oxidative Capacity

**Figure 5:**
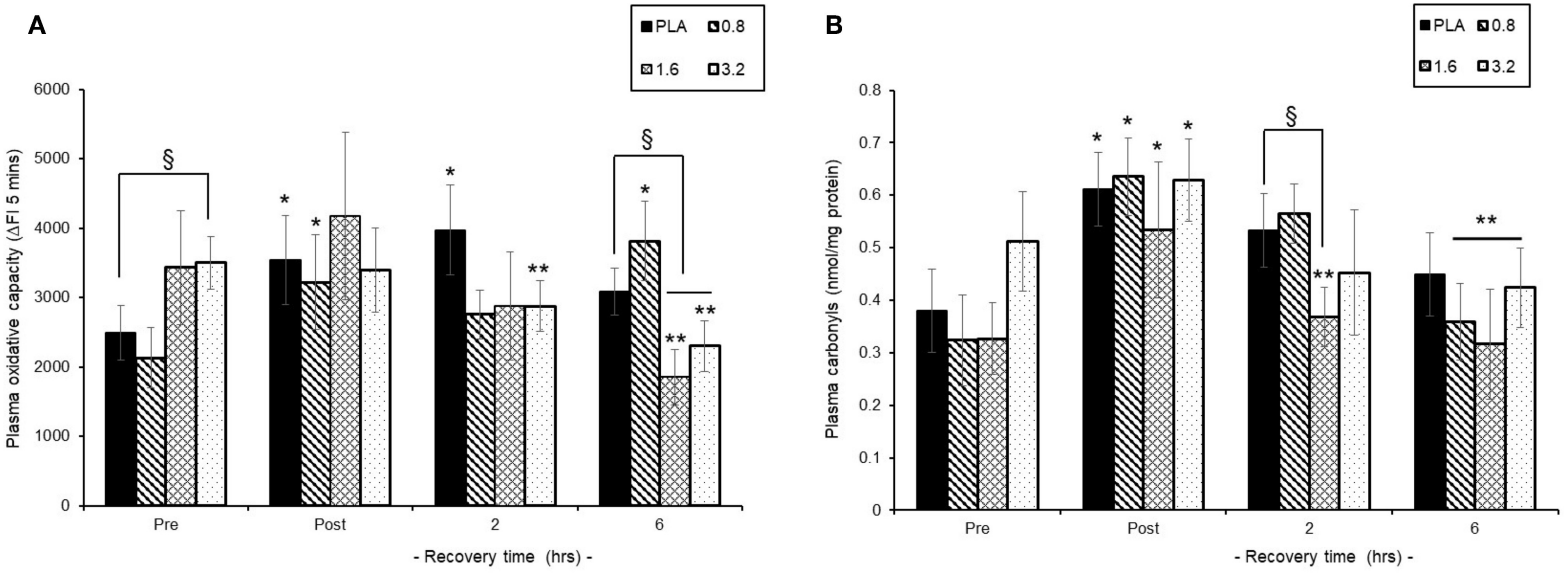
Ability of blackcurrant anthocyanin-rich extract (BAE, mg/kg) consumption 1 h prior to exercise to influence plasma oxidative capability **(A)** and protein carbonyl levels **(B)** during post-exercise recovery. Results are mean ± SEM, *n* = 8 individuals per group. ^*^P < 0.05 and ^**^P < 0.05 represents a statistical difference from pre- and post-exercise values, respectively, for each intervention group. ^§^*P* < 0.05 represents a statistical difference from placebo for the BAE groups highlighted.

Pre-exercise consumption of BAE had a differential influence on both baseline and post-exercise recovery plasma oxidative capacity (Figure 5A). Consumption of either PLA or 0.8 mg/kg BAE 1 h prior to exercise displayed similar plasma oxidative capability; 2491 ± 396 vs. 2129 ± 396 ΔFI_5mins_, respectively. In contrast, consumption of 1.6 or 3.2 mg/kg BAE prior to exercise showed elevated pre-exercise plasma oxidative capability compared to PLA; 3430 ± 817 or 3500 ± 379 ΔFI_5mins_, respectively. This increase in plasma oxidative capability within the 1.6 and 3.2 mg/kg BAE groups was still evident immediately post-exercise but by 2 h recovery was lower than post-exercise values_._ Furthermore, after 6 h post-exercise recovery, the plasma oxidative capability of participants in both the 1.6 and 3.2 mg/kg BAE intervention groups were significantly (*P* < 0.01) lower than their respective post-exercise values as well as those in the PLA group.

#### Plasma Protein Carbonyl Levels

Trial participants who consumed 0.8 or 1.6 mg/kg BAE prior to exercise showed a similar pre-exercise plasma carbonyl level to those in the PLA group (Figure 5B), whereas carbonyl levels in the 3.2 mg/kg BAE group was 37% higher. Plasma carbonyls measured immediately post-exercise were elevated (*P* < 0.05) in PLA (0.61 ± 0.07 nmol/mg protein), 0.8 (0.63 ± 0.07 nmol/mg protein) and 1.6 (0.53 ± 0.12 nmol/mg protein) mg/kg BAE groups and now similar to the levels measured in the 3.2 (0.63 ± 0.07 nmol/mg protein) mg/kg BAE group. However, by 2 h recovery, participants in the 1.6 and 3.2 mg/kg BAE groups showed a 32 and 27%, respective decline in post-exercise plasma carbonyl levels compared to those observed in the PLA group. After 6 h recovery, a decline in plasma carbonyls was observed in all intervention groups, with levels in the 0.8, 1.6, and 3.2 mg/kg BAE groups being significantly (*P* < 0.05) lower than post-exercise levels.

### Maintenance of Circulating Neutrophil Status and Function in Response to Exercise and Recovery

#### Circulating Neutrophil Population

**Figure 6:**
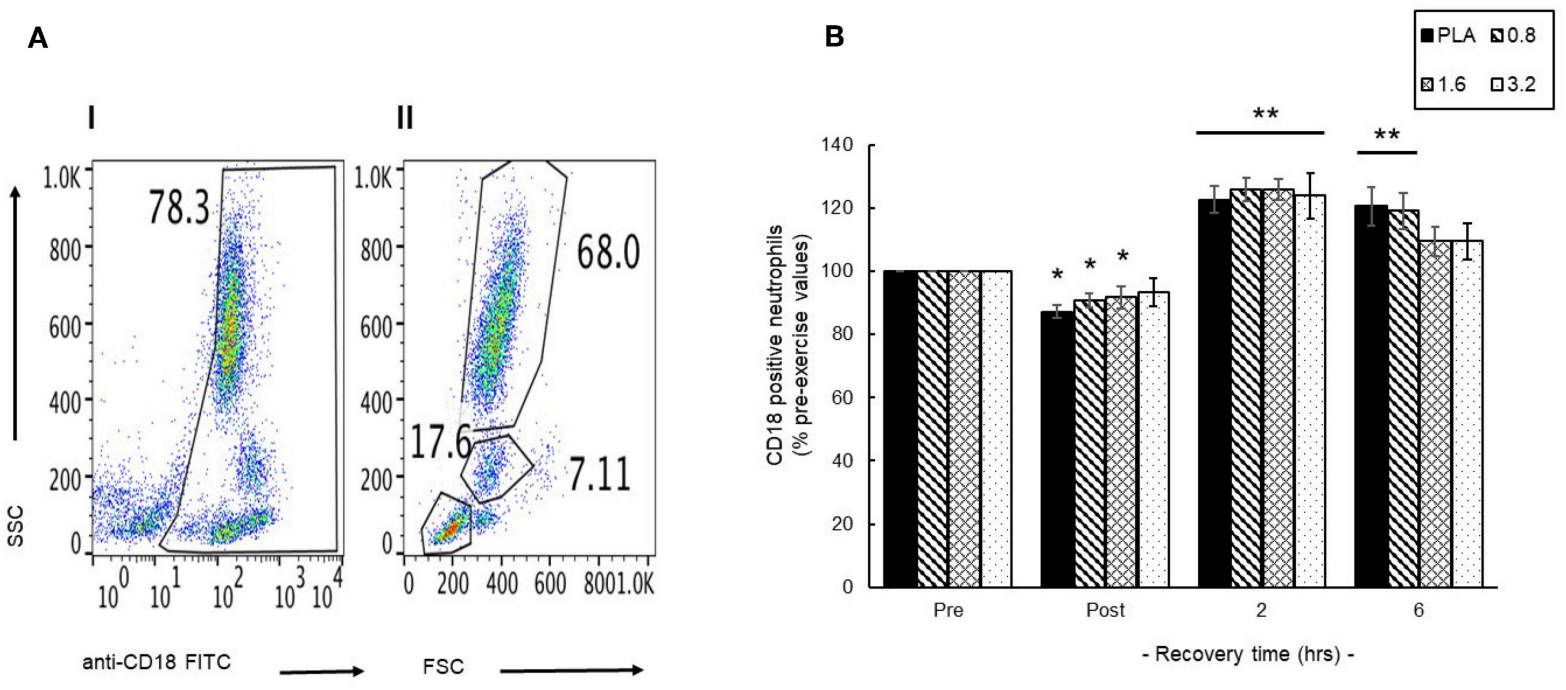
Identification of a blood CD18-labeled neutrophil population **(A)** and the influence of pre-exercise consumption of blackcurrant anthocyanin-rich extract (BAE, mg/kg) on the distribution of circulating neutrophils during exercise recovery **(B)**. Results are presented as either **(A)** scatter plots (I /II) highlighting selection of the CD18 neutrophil population or **(B)** mean ± SEM % pre-exercise values, *n* = 8 individuals per group. ^*^P < 0.05 and ^**^P < 0.05 represents a statistical difference from pre- and post-exercise circulating neutrophil values, respectively, for each intervention group.

An initial representative scatter plot (Figure 6A, I) of FITC-CD18 labeled leukocytes showed that 78% of the leukocyte population exhibited CD18 surface expression (CD18^+^). Further gating of this population enable separation of a discrete CD18^+^ neutrophil population; 68% (Figure 6A, II) that was then used to evaluate changes in the circulating neutrophil population. Pre-exercise consumption of BAE had no effect on the blood CD18^+^ neutrophil population and so changes during exercise recovery were evaluated as % pre-exercise levels (Figure 6B). A transient decline in circulating CD18^+^ neutrophils was observed immediately after exercise (post-exercise) in PLA, 0.8 and 1.6 mg/kg BAE groups. In contrast, by 2 h post-exercise recovery a significant (*P* < 0.01) increase in blood CD18^+^ neutrophils was observed in all treatment groups: PLA (23 ± 4%), 0.8 (26 ± 4%), 1.6 (26 ± 3%) and 3.2 (24 ± 7%) mg/kg BAE. This was still evident in the PLA and 0.8 mg/kg BAE groups after 6 h recovery, whereas circulating CD18^+^ neutrophil levels in participants who had consumed 1.6 or 3.2 mg/kg BAE had returned to pre-exercise values.

#### Neutrophil CR3^+^ Expression

**Figure 7:**
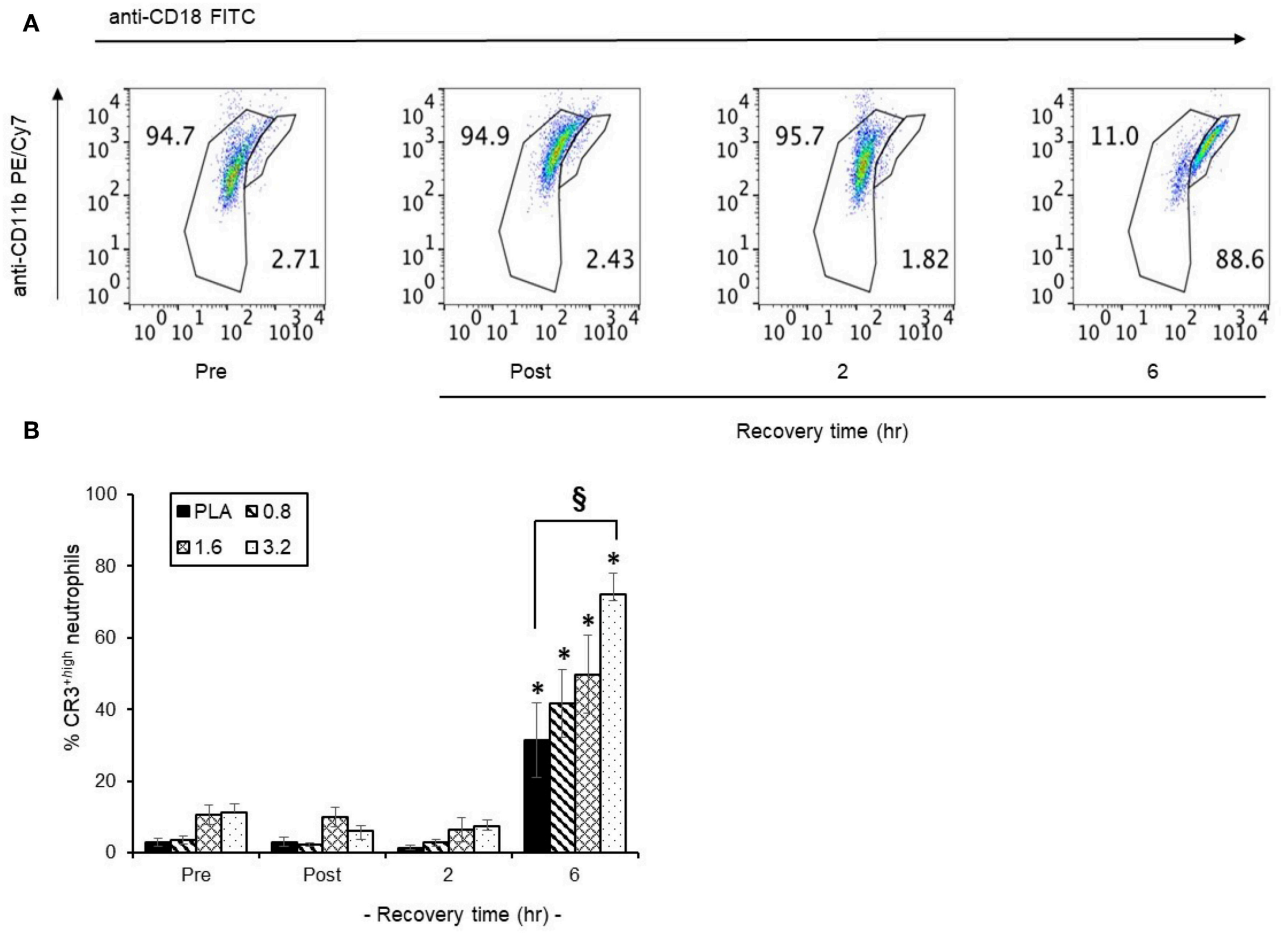
Influence of blackcurrant anthocyanin-rich extract (BAE, mg/kg) consumption 1 h prior to exercise on neutrophil complement receptor 3 (CR3^+^) surface expression during exercise recovery. Results are presented as CD18 neutrophil populations exhibiting CR3^+^ surface expression in the form of **(A)** a series of scatter plots or **(B)** mean ± SEM % CR3^+high^ neutrophils, *n* = 8 individuals per group, ^*^P < 0.01 represents a statistical difference from respective pre-exercise values for each intervention group. ^§^*P* < 0.01 represents a statistical difference from the placebo group.

The temporal distribution of CR3^+^ surface expression in circulating neutrophils in a representative scatter plot (Figure 7A) revealed that following 6 h post-exercise recovery, 88.6% of circulating neutrophils were CR3^+high^ compared to only 2.7% in pre-exercise blood neutrophils. Since CR3^+^ surface expression is important for neutrophil phagocytosis, we focused on the efficacy of timed BAE consumption on the expression profile of CR3^+high^ neutrophils in response to exercise and recovery (Figure 7B). A significant (*P* < 0.01) increase in the CR3^+high^ neutrophils was observed in the PLA group (31.3 ± 10.4 %) compared to pre-exercise levels (2.9 ± 1.0 %). This increase in CR3^+high^ neutrophils was also observed in BAE intervention groups, where a dose-dependent increase in circulating CR3^+high^ neutrophils compared to pre-exercise levels was observed (Figure 7B): In addition, pre-exercise consumption of 3.2 mg/kg BAE caused an increase in CR3^+high^ neutrophils (72.1 ± 5.7 %) that was greater (*P* < 0.01) than that observed in the PLA group.

#### Phagocytic Capability of Circulating Neutrophils

**Figure 8:**
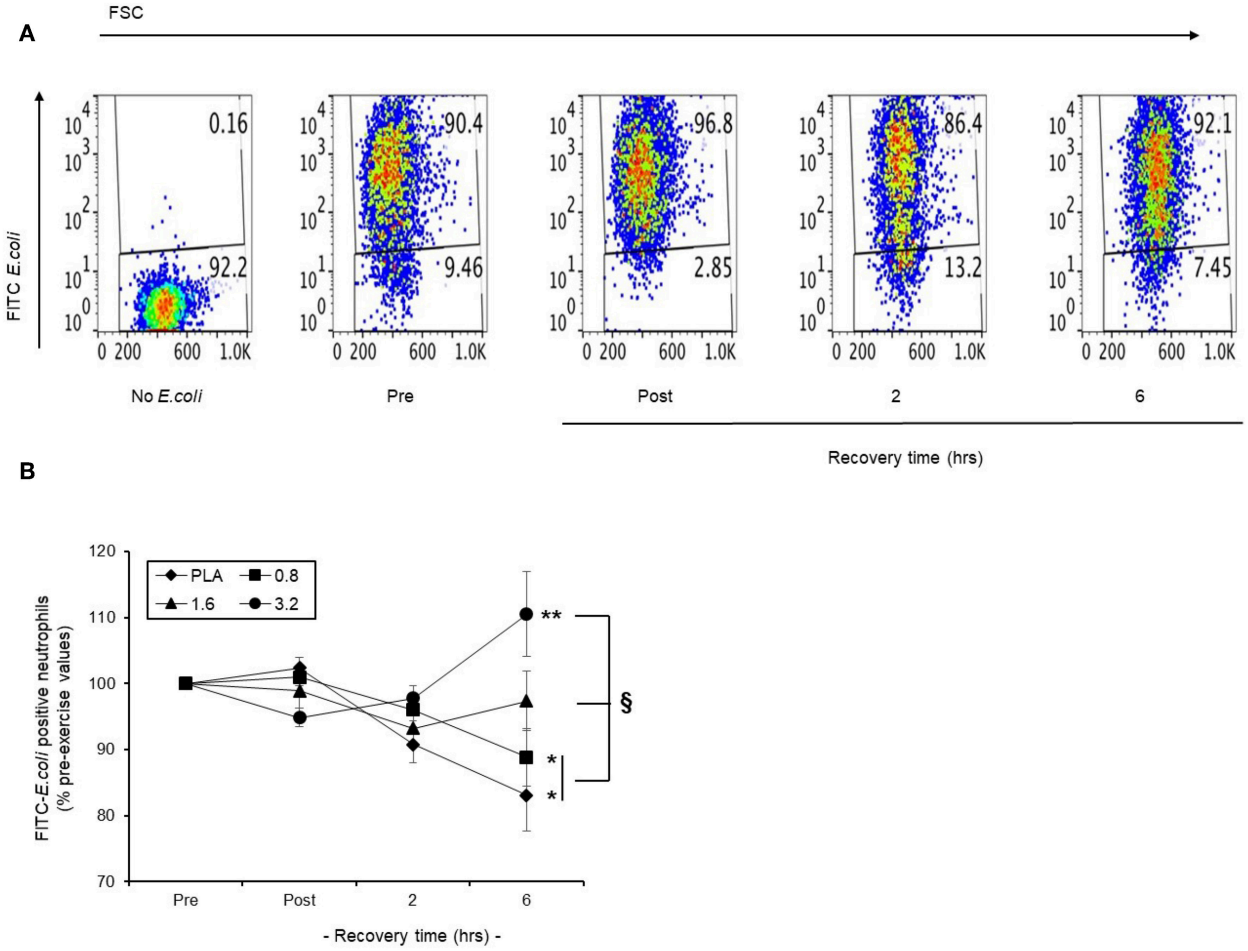
Capability of pre-exercise blackcurrant anthocyanin-rich extract (BAE, mg/kg) consumption to preserve neutrophil phagocytic activity during exercise recovery. Results are presented as the distribution of FITC-*E.coli* positive neutrophils in the form of **(A)** a series of scatter plots or **(B)** mean ± SEM % pre-exercise values, *n* = 8 individuals per group. ^*^P < 0.05 represents a statistical difference from pre-exercise values for each intervention group. ^§^*P* < 0.05 represents a statistical difference from the placebo group.

In a representative scatter plot (Figure 8A), we found that baseline phagocytosis (90.4 %) of FITC-*E. coli* by blood neutrophils became lower (86.4%) following a 30 min row, and returned to pre-exercise levels (92.1%) by 6 h recovery. We applied this model to examine the efficacy of pre-exercise BAE consumption on the phagocytic capability of circulating neutrophils during exercise recovery. Baseline blood neutrophil phagocytosis capability was similar in all intervention groups and so results are presented as % pre-exercise values (Figure 8B). In the PLA group, exercise caused a gradual decline in the ability of blood neutrophils to phagocytosis opsonised FITC-*E. coli* during recovery, which by 6 h was 17% (*P* < 0.05) lower than pre-exercise values. Pre-exercise consumption of BAE exhibited a dose-dependent ability to preserve neutrophil phagocytic capability during post-exercise recovery. Whilst a 12% (*P* < 0.01) drop in blood neutrophil phagocytic capability was observed 6 h post-exercise in the 0.8 mg/kg BAE group, no decrease was observed in the 1.6 or 3.2 mg/kg BAE intervention groups: By 6 h post-exercise recovery, blood neutrophil phagocytosis capability within these intervention groups was still 97.4 ± 4.4 and 110.5 ± 6.4 % pre-exercise values, respectively, and a significantly different (*P* < 0.05) phagocytosis capability was observed in the PLA and 0.8 mg/kg BAE groups.

## Discussion

The data reported here as a whole demonstrate that a single, timed consumption of BAE dose-dependently prior to exercise supported the recovery from exercise-induced oxidative stress, and maintained neutrophil function, thus reducing the risk for opportunistic infections. Previous studies show that the consumption of anthocyanin rich foods/supplements results in a rapid increase in plasma anthocyanin concentration, usually peaking 1–2 h post-consumption (47, 48). This early discrete plasma bioavailability profile enables the nutritional efficacy of these anthocyanins to be evaluated in short-term intervention studies. In this current study we found a time-dependent increase in plasma anthocyanins following the consumption of an anthocyanin extract from 3.2 mg/kg total anthocyanins that peaked at 2 h. In contrast, Matsumoto et al. (49) reported that plasma anthocyanin levels after the consumption of a New Zealand blackcurrant extract (containing 17 mg/kg anthocyanins) peaked at 1 h post-consumption. The temporal bioavailability profile of the plasma anthocyanins and their concentrations were similar to our findings here. The differences in the time of the peak in plasma anthocyanin levels observed between these two studies is unclear and may be due to a number of factors including the different chemical anthocyanin compositions of the extracts, plasma anthocyanin extraction and analyses methods used, the amount of anthocyanins consumed by trial participants, and variation in the absorption kinetics by different participant cohorts (47). In our study, the peak plasma anthocyanin bioavailability at 2 h post-consumption was similar in both studies irrespective of the initial blackcurrant extract dose consumed. However, despite the reported anthocyanin bioavailability differences, the findings from our current study and that of others (36, 49) demonstrate a close relationship between optimal plasma anthocyanin bioavailability and functional efficacy.

It is still debatable whether or not consuming foods that exhibit powerful inherent antioxidant properties actually results in increased human plasma antioxidant capacity that is capable of attenuating oxidative stress resulting from exercise (2). In this pilot study, we found that the consumption of the BAE had no impact on overall plasma antioxidant capacity prior to, or during recovery from, a 30 min rowing exercise at 70% VO_2_max. Our preliminary findings are supported by Morillas-Ruiz et al. (50), who demonstrated that the ingestion of a fruit drink enriched in polyphenolics 15 min prior to a 90 min cycle (70% VO_2_max) had no effect on overall plasma antioxidant status despite mediating a significant decrease in post-exercise plasma protein carbonyls. These results indicate that the facilitated recovery from exercise-induced oxidative stress is unlikely to be due to the inherent antioxidant properties of the ingested anthocyanin extract. Others have shown that similar plasma levels of anthocyanins did not exhibit any antioxidant properties in chemical and *in vitro* antioxidant studies (22, 23). However, Matsumoto et al. (51) showed that the consumption of a blackcurrant extract containing 33 mg/kg of anthocyanins, exhibited similar plasma anthocyanin concentrations to that found in our current study, and was able to mediate a detectable increase in plasma antioxidant capacity; although a different chemical assay, compared to the one we used in this current study, was used to detect the changes in plasma antioxidant capacity. Alternatively blackcurrant anthocyanins may activate cellular redox-sensitive mechanisms that up-regulate antioxidant systems to increase antioxidant capacity (20, 25, 50). In this pilot trial, we observed an increase in plasma oxidative capacity 1 h following consumption of either 1.6 or 3.2 mg/kg BAE (Figure 4A) prior to exercise that was greater than the placebo group. Since anthocyanins and their metabolites exhibit electrophilic properties (52), it is feasible that the pre-exercise increase in plasma oxidative capability observed may be the consequence of an increase in plasma anthocyanins. This in turn may lead to the activation of cellular redox-sensitive processes, such as nuclear redox factor-2/antioxidant response element (Nrf2/ARE) transcription, and the up-regulation of cellular antioxidant systems that contribute to the lower plasma oxidative stress indices observed during recovery. Berryfruit anthocyanins in particular, have been shown to activate a number of cellular transcription factors, especially the Nrf2 pathway that up-regulate the expression of a number of redox-sensitive cellular defense pathways including redox-sensitive antioxidant enzymes (e.g., HO-1, NQO1) to enhance overall cellular antioxidant capacity (53, 54). Other plant-derived polyphenols have been shown to activate cAMP-respective element (CRE) signaling, which mediates the up-regulation of the master antioxidant reduced glutathione (GSH) (55). It is thought that certain anthocyanin glycosides and/or primary metabolites such as protocatecheuic acid and/or gallic acid indirectly activate cellular Nrf2/ARE via the chemical conversion of diphenols to quinines through auto-oxidation to an electrophile quinine-type molecule or reduce proteasome ubiquitination activity that in turn causes fluctuations in oxidative and/or redox status (52, 56–58). Moreover, recent cell experiments conducted within our group found that blackcurrant anthocyanins enhance tBHQ- and redox (copper II)-activation of Nrf2/ARE transcription using an ARE-luciferase reporter HepG2 cell-line and up-regulate antioxidant enzymes, HO-1 and thioredoxin reductase (data not shown). Our findings support Thoppil et al. (30) who reported in a rat hepatocellular carcinoma study that daily consumption of blackcurrant anthocyanins (100 or 500 mg/kg) for 18 weeks increased the antioxidant capacity via the stress-induced activation of Nrf2/ARE. It is therefore feasible that the auto-oxidation of bioavailable blackcurrant-derived anthocyanins, either alone or in conjunction with exercise-induced ROS (or redox fluctuations), leads to the activation of cellular stress-sensitive signaling mechanisms, such as Nrf2/ARE, enabling the up-regulation of cell antioxidant capacity and the improved recovery from exercise-induced oxidative stress observed in this initial study.

Maintaining functional innate immunity in response to exercise-evoked oxidative stress and acute inflammation, especially during post-exercise recovery, is vital to limiting susceptibility to opportunistic pathogen infection (10–12, 59). Neutrophil recruitment in response to exercise and during recovery is related to a number of factors, including haemodynamic changes, increased cardiac output, circulating stress hormones (e.g., adrenaline) or increased inflammation-driven chemokines, e.g., IL-8, GM-CSF (60–62). This tightly coordinated event creates a dynamic neutrophil population that responds to inflammatory status and so nutritional strategies designed to facilitate exercise recovery, also need to support (and not suppress) flexibility in circulating neutrophil numbers (12). In this pilot study, we found timed consumption of BAE (3.2 mg/kg) prior to exercise attenuated the transient post-exercise decline in circulating neutrophils as well as supported the increase in circulating neutrophils during recovery. Although the underlying mechanism is unknown, it is feasible that the bioavailability of blackcurrant anthocyanins prior to exercise may improve blood flow (36) to support rapid neutrophil influx during recovery (outflux after recovery) and/or maintain the existing neutrophil population through the removal of potential oxidants such as lactic acid (63). Since neutrophils are the first line of defense against microbial infection, it is also important that circulating neutrophils possess and maintain their functional capacity in response to exercise and subsequent recovery. The functionality of circulating neutrophils in response to exercise is influenced by fluctuations in intracellular ROS—redox status (64, 65) and neutrophil phagocytic function in response to exercise and during recovery is dependent upon the intensity and duration of the exercise and the fitness of the individual (66–68). The 30 min row at 70% VO_2_max employed in our study caused a time-dependent decrease in blood neutrophil phagocytosis (opsonised *E. coli*) capability during post-exercise recovery. A similar decrease in neutrophil function has been observed in other exercise models (59, 65–67), with is being attributed to exercise-induced oxidative damage within the neutrophil, resulting in a loss of antioxidant defense mechanisms causing defective chemotaxis and phagocytosis. Here, in this preliminary study, we found that the consumption of the BAE (1.6 or 3.2 mg/kg) 1 h prior to exercise prevented the decline in neutrophil phagocytic capability. The master antioxidant regulator, Nrf2 is highly expressed in neutrophils enabling the leukocyte to respond quickly to changes in oxidative and redox status by up-regulating its antioxidant status (69). Since plant polyphenols, including anthocyanins (20, 25, 50) activate cellular Nrf2/ARE transcription (direct or indirect mechanism), it is conceivable that by timing the plasma bioavailability of blackcurrant anthocyanins prior to exercise may facilitate the up-regulation of neutrophil antioxidant defenses (i.e., priming event) to preserve functionality against exercise-induced oxidative stress, however a large study is required to support this hypothesis. This notion, however, is supported in a study by Suzuki (65) who suggested that certain polyphenols may enhance neutrophil antioxidant capacity leading to a retention of their functionality. Furthermore, acute exercise has been shown to influence neutrophil function via altering functional membrane receptor profiles of circulating neutrophils (60, 61, 66, 70). Complement receptors (CRs), CIqR, CR1, CR3 (CD11b/CD18) and CR4 (CD11c/CD18), expressed on the surface of neutrophils efficiently recognize microbes bound with complement components. These surface receptors are sensitive to fluctuations in exercise-induce changes in oxidative and redox status, which can result in either a down-regulation or membrane cleaving resulting in loss of neutrophil function (71). Since CR3 expression is critical for phagocytosis it becomes important to maintain expression levels of CR3 during exercise recovery to minimize susceptibility to infection. In our current pilot study, we found the consumption of BAE caused a dose-dependent enhancement in the surface expression of CR3 during post-exercise recovery, which paralleled the increased phagocytosis capability observed 6 h post-exercise. These preliminary findings are supported by Pizza et al. (72) who demonstrated that neutrophil and monocyte CR3 and CD64 expression together with neutrophil functionality fluctuated over 96 h during adaptation to eccentric exercise; an increase in post-exercise neutrophil CR3 was observed after 6 h recovery following the first eccentric exercise bout. In contrast, Gavrieli et al. (68), found no change in neutrophil CR3 surface expression 24 h after the completion of a 30 min treadmill run (75% VO_2_max) in physically active males. Despite the exercise-induced differences in neutrophil receptor/function relationship, regular exercise, especially as we age (70), is generally shown to preserve circulating neutrophil numbers and function, including maintaining the surface expression of CR3, for appropriate anti-microbial chemotaxis, phagocytosis and oxidative burst function. Whilst our current findings suggest that timing the bioavailability of blackcurrant anthocyanins prior to a moderate exercise (i.e., consuming ≥ 1.6 mg/kg BAE 1 h prior to exercise) preserves innate neutrophil immunity reducing the susceptibility to opportunistic microbial infection during post-exercise recovery, a larger human study is required to define efficacy.

In conclusion, it is becoming apparent that the beneficial outcomes of anthocyanin supplementation on innate immunity (including alleviating oxidative stress), may be tailored to an individual's needs. Nutritional studies report that regular consumption of foods high in polyphenols protect against and alleviate the inflammatory symptoms of cardiovascular disease, type 2 diabetes, obesity and other inflammation-driven diseases (73, 74). Further, timed consumption of these foods/supplements, demonstrated in this study and by others (25, 33, 75) can support exercise recovery and maintain natural immune defenses, which is independent of the food products inherent antioxidant property. Although the cellular mechanisms are currently unconfirmed, it is most likely to involve the modulation of innate immune systems (via the activation of cellular stress and/or redox sensitive processes) such as antioxidant; Nrf2/ARE (53), inflammation regulation; *Nuclear factor (NF)*-κ*B*, Activator protein-1 (AP-1) (34, 73, 76) and/or immune homeostasis; Forkhead box class O (FOXO) (77) signaling, rather than through the direct antioxidant properties of anthocyanins. Moreover, the potential adaptive action of plant polyphenols to maintain innate immunity is supported by others (20, 25, 50), including a study by McAulty et al (75) who found that the consumption of blueberries 2 h prior to exercise facilitated recovery from exercise-induced oxidative stress and natural killer cell function. Our preliminary findings support the inclusion of appropriately timed and dosed nutritional intervention of ≥ 1.6 mg/kg BAE to aid post-exercise recovery and contributes to on-going meta-analysis collection that pre-exercise consumption of anthocyanin-rich foods may facilitate exercise recovery from oxidative stress and preserve innate immune function.

## Ethics Statement

The human trials conducted in this study were approved by either the Northern (NTY/09/10/105, Hamilton) or Central (CEN/09/11/088, Wellington) Ethical Regional Committees of New Zealand.

## Author Contributions

RH, KL, and SH designed the study and gained human ethical approval from Human Disability Ethics Committees (HDEC). RH, KL, and RW recruited the participants and performed the human trials. SH, KL, JR, AP, RW, JC, and DJ carried out the experimental analysis on collected blood samples and the interpretation of results. KL, SH, and RW developed and performed the biochemical assays. JR and AP designed, performed, and analyzed flow cytometry experiments. JC and DJ developed and performed the anthocyanin LC-MS analysis. RH, NB, and SH drafted the manuscript. JR, AP, and JC revised the manuscript critically for important intellectual content. SH approved the final version of the manuscript submitted.

### Conflict of Interest Statement

The authors are employees of the New Zealand Institute for Plant and Food Research Ltd which has no royalty agreement associated with sales of New Zealand blackcurrant products. The authors declare that there is no individual personal financial relationship. The data sets for this manuscript are not publicly available because of commercial sensitivity. Requests to access the datasets should be directed to the corresponding author.
